# Promoter Methylation Status Modulate the Expression of Tumor Suppressor (RbL2/p130) Gene in Breast Cancer

**DOI:** 10.1371/journal.pone.0134687

**Published:** 2015-08-13

**Authors:** Farman Ullah, Taimoor Khan, Nawab Ali, Faraz Arshad Malik, Mahmood Akhtar Kayani, Syed Tahir Abbas Shah, Muhammad Saeed

**Affiliations:** 1 Cancer Genetics and Epigenetics Lab, Department of Biosciences, COMSATS Institute of Information Technology (CIIT), Islamabad, Pakistan; 2 Department of Biotechnology and Genetic Engineering, Kohat University of Science and Technology (KUST), Kohat, Pakistan; University of Navarra, SPAIN

## Abstract

**Background:**

Aberrant expression of tumor suppressor genes may correspond to the abnormal cell development and tumorigenesis. Rbl2/p130, a member of retinoblastoma family of proteins, has growth suppressive properties. Numerous studies reported de-regulation of Rbl2/p130 in various types of cancer as a consequence of a number of genetic alterations. However, role of epigenetic mechanisms like DNA methylation in Rbl2/p130 expression remains elusive.

**Methods:**

In the current study, 76 breast cancer tumors along with normal tissues (n = 76), blood (n = 76) of respective individuals and control blood (n = 50) were analyzed. Rbl2/p130 expression was analyzed by quantitative real time PCR (syber green method). Promoter methylation status was studied through methylation specific PCR of bisulfite converted genomic DNA. Data was analyzed using various statistical tests.

**Results:**

We report significantly reduced Rbl2/p130 expression (P = 0.001) in tumors tissues as compared to control samples. Similarly, Rbl2/p130 expression varies with age and disease stages (P = 0.022), which suggest its involvement in tumor progression. Aberrant promoter methylation (Δmeth) was found in almost all the diseased samples and that was significantly different (P<0.001) with control samples. Similarly, methylation status varies significantly with tumor progression stages (P = 0.022). Hyper-methylation was observed at -1, +3, +15 and +75 of Rbl2/p130 promoter flanking around the TSS. Statistical analysis revealed that Rbl2/p130 expression negatively correlates to its promoter methylation (r = -0.412) in tumor tissues. Our results reflect an epigenetic regulation of Rbl2/p130 expression in breast cancer. This highlights the importance of Rbl2/p130 promoter methylation in breast cancer pathogenesis.

## Introduction

The eukaryotic cell cycle is a precisely regulated set of events, which ensures cell growth and precise transmission of genetic information to daughter cells. Alterations in the activity of genes regulating cell cycle, leads to abnormal cells division and proliferation [1]. Such deregulations are common in cancer pathogenesis[2].

Members of retinoblastoma (Rb) family of proteins play pivotal role in cell cycle regulation. Rb family of proteins comprises of three members namely retinoblastoma protein (pRb/p105), retinoblastoma like protein-1 (Rbl1/p107) and retinoblastoma like protein-2 (Rbl2/p130). These three members of the family are collectively referred to as “pocket proteins”[3–5]. The term “pocket proteins” derives from conserved pocket domain present in all three members of the Rb family through which they bind to viral oncoproteins [4, 6, 7]. Rb proteins regulate cell cycle progression by associating themselves with E2F family of transcription factor. Phosphorylation of Rb proteins by CDK/Cyclin complexes disrupts these complexes, resulting in the evasion of G1/S checkpoint. Recently acetylation of Rb proteins has also been reported *in vivo* and in tumor tissues [8].

Among growth suppressive properties of Rb family members, Rbl2/p130 has shown suppression of tumor growth *in vivo* [9], suggesting its protective effects against cancer. Delivery of wild-type Rbl2/p130 gene may revert malignant phenotype in various cancer types [10] including human cervix carcinoma (C33A)[11]; T98G human glioblastoma [9]; nasopharyngeal carcinoma and Saos-2 human osteosarcoma cells[3, 9]. Due to the presence of E2F4-p130 complexes in abundance in quiescent cells, some authors have proposed E2F4-p130 complexes as marker of Go phase of the cell cycle [3, 12]. De-regulated expression of Rbl2/p130 gene has also been reported in lung [13], endometrial [14] and ovarian cancers [15, 16].

Change in gene expression of Rbl2/p130 can be related to either genetic or epigenetic mechanisms. Genetic mechanisms include gene variation, transcript stability and changes in regulatory sequences (promoter), whereas epigenetic mechanisms include DNA methylation at regulatory sequences, chemical modification of DNA binding proteins, and remodeling of chromatin architecture. There is an accumulating body of evidence linking de-regulated expression of Rbl2/p130 to numerous of genetic factors [17]. However very little is known about epigenetic mechanisms regulating Rbl2/p130 expression.

Epigenetic modifications play a critical role in cancer initiation and progression [18]. Aberrant promoter methylation at CpG sites are critical epigenetic tags that may regulate gene expression in eukaryotic cells [19, 20]. Generally promoter methylation relaxes chromatin structure that may alter the expression of cell cycle regulatory genes [21]. Aberrant promoter methylation of tumor suppressor genes that down regulate the expression level in various cancer types have been reported such as thyroid hormone receptor β1 (TRβ1) [22] and VEZT gene in gastric cancer[23], N-myc downstream-regulated gene 2 (NDRG2) in colorectal cancer[24], BRCA1 and TUSC5 genes in breast cancer[25], and von Hippel–Lindau (VHL) gene in kidney[26] and multiple myeloma[27]. Recently hyper-methylation at pRb promoter in head and neck cancer has also been shown to play role in tumor progression [28]. Although it’s down regulation has been reported in number of cancers but the status of its promoter methylation remains elusive.

The focus of the current study is to analyze Rbl2/p130 mRNA transcript expression and its promoter methylation in breast tumor and control tissues, to assess their role in breast cancer carcinogenesis. These finding will later correlated with clinico-pathological feature of the cohort to assess the role of Rbl2/p130 gene in cancer development.

## Materials and Methods

### Identification of Patients and Tissue Samples Collection

Fresh tissue (n = 76) and their adjacent normal control tissues (ANCT) (n = 76) along with blood sample (n = 76) from the same patients were collected from local hospitals at the time of surgery. ANCT control samples were selected microscopically, with uninvolved areas more than 2 cm away from the tumor areas based on clinical observations made by oncologist. Tissue samples were collected and preserved in RNA Later solution to avoid RNA degradation, whereas blood samples were collected in EDTA tubes. Moreover blood samples from healthy individuals (n = 50) were also collected. The inclusion criterion for the control samples was the absence of a prior history of cancer or a pre-cancerous lesion. Patients and controls suffering from familial diseases (i.e., diabetes, blood pressure and cardiovascular impairment) were excluded from this study. Samples were collected from the patients after signing the informed consent during their clinical visits immediately after surgery, from Holy Family Hospital Rawalpindi, Pakistan Institute of Medical Sciences Islamabad, Leady Reading Hospital Peshawar and Khyber Teaching Hospital Peshawar. Tumor tissues and adjacent normal tissue were confirmed by an oncologist. All the samples were stored at -20°C until further analysis. This study was conducted with prior approval from ethical committees of both CIIT and collaborating hospitals. Patient were categorized into two age groups, first cohort comprised of patients below the age of 45(<45) years, and the 2^nd^ cohort included patients with age of 45 or greater than 45 years (≥ 45).

The current study was conducted on 76 breast cancer patients including tumors their normal control, and blood of respective individuals. Majority of the patients belonged to Punjab and Khyber-Pakhtunkhwa province. Patient’s data was evaluated on different parameters e.g. age, tumor staging and grade. ~43% of the patients belonged to 1^st^ study cohort that was below the age of 45(<45) years, whereas ~57% of the patients belong to 2^nd^ cohort (of or greater than the age of 45 years; ≥ 45). Among them ~64% cases were premenopausal and the remaining were postmenopausal. Other demographic and clinical characteristics of the cancer patients included in this cohort are mentioned in the Table 1.

**Table 1:** Demographic and Clinico-Pathological Characteristics of Breast Cancer Cohort.

Study Variables		N (%)	Frequency of Study Cohort I (<45 Years)	Frequency of Study Cohort II (≥ 45 Years)
**Menopausal Status**	Pre-menopausal	49 (64.47)	44	5
	Post-menopausal	27 (35.53)	0	27
**Histological Types**	IDC	56 (73.68)	15	41
	ILC	16 (21.06)	2	14
	DCI	4 (5.26)	2	2
**Disease Stages**	Stage I	37 (48.68)	22	15
	Stage II	22 (28.95)	5	17
	Stage III	14 (18.42)	6	8
	Stage IV	03 (3.95)	1	2
**Disease Grading**	Well Differentiated (G1)	34 (44.74)	2	32
	Moderate Differentiated (G2)	28 (36.84)	7	21
	Poor Differentiated (G3)	14 (18.42)	9	5

### RNA Extraction and Real Time PCR Quantification

RNA extraction from tissues (tumor + ANCT) was carried out using standard Trizol method [29] with slight modifications as per requirement. RNA was stored and at -80°C until further analysis. Reverse transcription polymerase chain reaction (RT-PCR) was carried out using super script first-strand cDNA synthesis kit (Invitrogen, USA). Gene specific primers for Rbl2/p130 (Left primer; AGAGGATGCTGAGGAGGAAA, Right primer; CAATAGCCTGGGTTGGATCT) and β-actin (internal control) (Left primer; CACTCTTCCAGCCTTCCTTC, Right primer; TGATCTCCTTCTGCATCGTG) were used for quantitative real time PCR analysis using Syber dye base approach. qPCR was performed on Step One Plus Real-Time PCR system (Applied Biosystems). Thermo-cycler conditions were 95°C for 10 min (initial denaturation) followed by 40 cycles of 95°C for 15 seconds, 54°C for 60 seconds and 72°C for 20 seconds with a final extension of 72°C for 1 min and the results were interpreted using their delta delta ct values.

### DNA Extraction and Bisulphite Modification

DNA extraction from tissue (tumor and ANCT) as well as blood samples were performed separately using standard phenol-chloroform method [30] and stored at -20°C for further use. 200–500 ng of genomic DNA was used for bisulphite conversion of un-methylated cytosine `C`into thymine `T`using commercially available EpiJET bisulfite conversion kit (Cat ≠ K 1461) as per manufacturer’s instructions.

### Primer design for Methylation Analysis

A set of DNA methylation independent primers (Left Primer; GGTGGGGAGGTACGTGTTTA, Right Primer; GCCACAGTCCTGATCCTAGT) were designed for ~1.2 kb fragment (NCBI,53434268–53435426) flanking around Rbl2/p130 TSS (transcription start site) using primer blast online tool [31]. These primers were designed from a region where CpG’s contents were less. Moreover, for detection of DNA methylation status a pair of methylation/un-methylation specific primers (Left Meth; TTTGAATCGTCGGTTTGGATTAC, Right meth; CTCGAAACGACTAAAAACCTCGAA, and Left Un-Meth; TTTTTTTGAATTGTTGGTTTGGATTATG, Right Un-Meth; TTAACCCTCAAAACAACTAAAAACCTCA) around TSS of Rbl2/p130 was designed using MethPrimer online tool[32].

### Methylation Specific PCR (MSP) and Sequencing

The targeted promoter region around Rbl2/p130 TSS was first amplified from bisulphite converted genomic DNA using methylation independent primers. Bisulphite converted genomic DNA (~100 ng) was amplified using following cycle conditions; 98°C for 30 sec (initial denaturation) followed by 35 cycles at 98°C for 10 sec, 62°C for 30 sec and 72°C for 30 sec; and a final extension at 72°C for 7 minutes. The amplified product (1 μl, ~ 100 ng) was used as a template in methylation-specific PCR (MSP) reaction. MSP was performed to detect promoter methylation status of Rbl2/p130 using a set of each methylation and un-methylation specific primers. Maxima Hot Start Taq DNA polymerase (Thermo Scientific Cat ≠ EP0601) was used to amplify MSP primers through given cycle conditions; 98°C for 3 min (initial denaturation) followed by 35 cycles at 98°C for 45 sec, 64°C for 40 sec and 72°C for 45 sec; and a final extension at 72°C for 7 minutes. CpG methylated human genomic DNA (Cat ≠ SD1131) from Thermo scientific was used as positive control for methylation reaction, whereas PCR water was used as negative controls for methyl specific reaction. For un-methylated-specific PCR unconverted DNA (without bisulphite treatment) was used as positive control. Moreover, amplified MSP products were sequenced by Macrogen (Korea) and the results were analyzed using BioEdit and ClastalW alignment tools.

### Gel Electrophoresis and Quantification

The PCR products from the above reactions were visualized on 2% agarose gel under UV illuminator (BioDoc Analyze, Biometra) and the relative band intensities were quantitated using densitometer to compare the degree of methylation and un-methylation quantitatively. Moreover, for the sake of convenience change in methylation status (Δmeth) was calculated by subtracting unmethylation values of a particular sample from its methylation values considering unmethylation as default condition.

### Data Analysis

Statistical analysis were performed with OriginPro 2015 (OriginLab, Northampton, MA). For analysis transcript profiles, expression data of the target gene (Rbl2/p130) was normalized against internal control gene (β-actin). The correlation among different factors was assessed by Pearson Correlation Coefficient test. Depending on experiment, the statistical significance was determined on 95% confidence intervals (CIs) using the Mann-Whitney test, an analysis of variance (ANOVA) and specific comparisons were made by Tukey’s honest significant difference (HSD) test. The values of P<0.05 were considered as significant. Statistical analysis enabled to investigate the correlation of methylation frequency and expression level of RbL2/p130 gene.

## Results

### Rbl2/p130 Expression is Significantly Lower in Tumor Tissues

**Fig 1:**
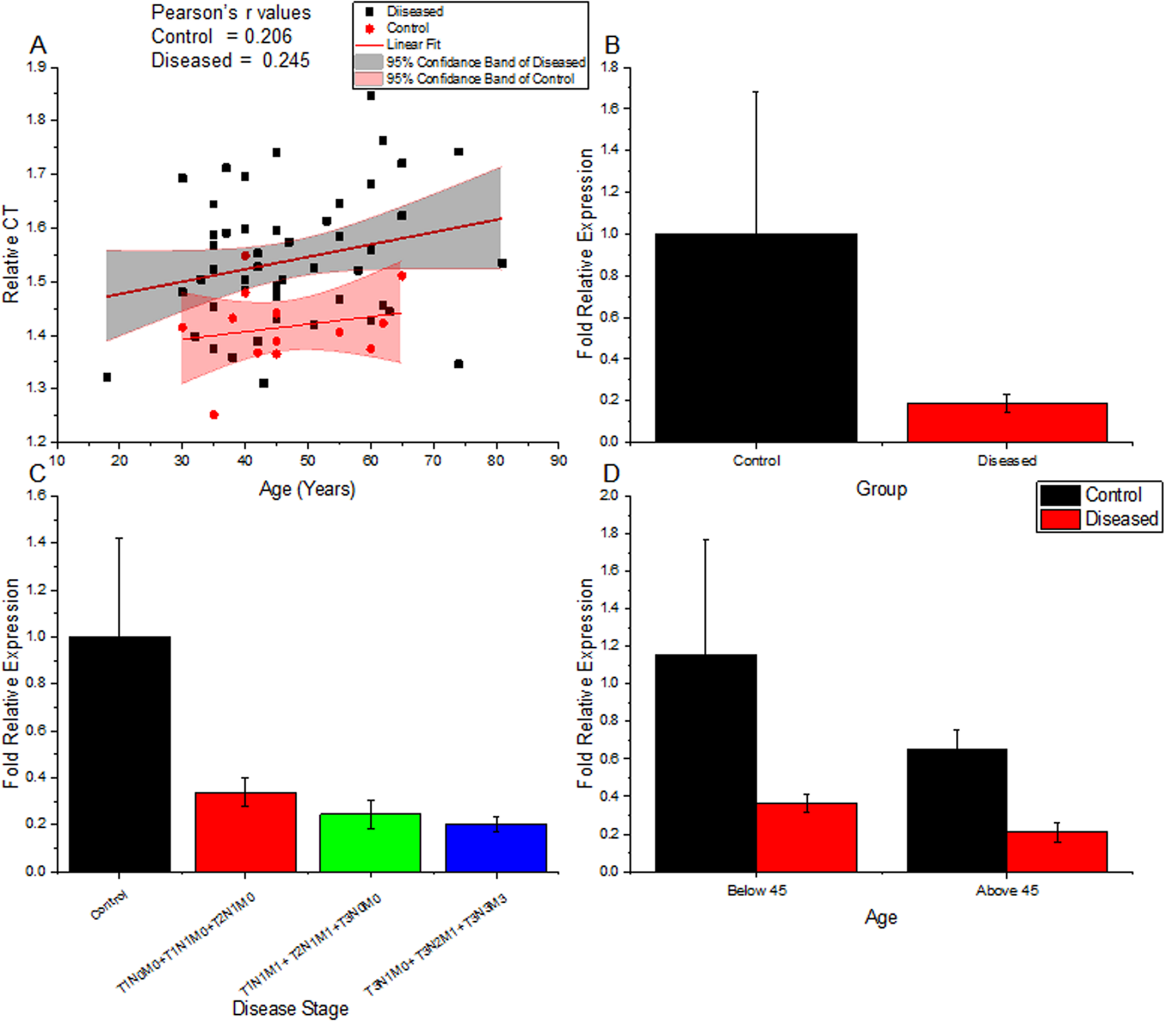
Relative expression profiling of Rbl2/p130 gene in different age groups and tumor stages. (**A)** Changes in relative Rbl2/p130 expression (ΔCT values) with age, both in normal and tumor tissues; (**B)** Observed changes in Rbl2/p130 in tumor and control tissues; (**C)** Differences in relative expression of Rbl2/p130 transcript as observed among control and different disease stages (**D)** Showing Rbl2 expression in control and disease samples, in both the study cohort (<45 and ≥45 years).

Using quantitative real-time RT-PCR, gene expression levels of Rbl2/p130 were determined in breast tumors and adjacent control tissues. The relative expression of Rbl2/p130 in breast tumors was found to be significantly lower (P = 0.001) than control samples in all age groups (Table 2 and Fig 1). This highlights the significance of Rbl2/p130 expression as an early prognostic tool for cancer pathogenesis. Moreover, levels of Rbl2/p130 were also found lower (P = 0.022) in stage or grade specific manner (Table B in S1 File). Patients with advance tumor stages (metastatic) had statistically significant low expression than patients with relatively early stages of tumor (Table 2 and Table A in S1 File). This observation suggests its involvement in tumor progression. Our results also suggest a decrease in Rbl2/p130 expression with growing age, but these differences are more pronounced in cancer patients (Fig 1A) where Rbl2/p130 expression values were more dispersed at older age. Similarly, the two-study cohort constituted on the basis of age (i.e. <45 and ≥45 years) also showed significant differences in Rbl2/p130 expression. In both age groups reduced Rbl2/p130 expression was recorded compared to their controls (Fig 1D; P = 0.001), although the decrease in expression was drastically low in patients below the age of 45 compared to their controls.

**Table 2:** Statistical Analysis of Rbl2/p130 Transcript Expression & Promoter Methylation Status among Tumor and Control Tissues of Breast Cancer Patients.

Variables	Category	Analysis	Samples	N	Mean	SD	SEM	95% CI of Mean		Skewness	P value
								Lower	Upper		
**Overall**	Overall	Expression	Control	13	1.00	1.52	0.42	0.08	1.92	3.34	0.001
			Diseased	50	0.28	0.24	0.04	0.21	0.35	1.64	
		Δmeth	Control	14	2880.94	504.44	134.82	2589.68	3172.19	-0.342	< 0.001
			Diseased	32	500.90	446.57	78.94	339.89	661.91	1.323	
**Age**	<45	Expression	Control	09	1.15631	1.8272	0.609	-0.24816	2.561	2.788	0.001
			Diseased	29	0.360	0.25	0.05	0.27	0.46	1.13	
		Δmeth	Control	04	3357.37	292.61	146.31	2891.76	3822.98	-0.048	0.019
			Diseased	05	452.62	197.04	88.12	207.96	697.27	-2.000	
	≥ 45	Expression	Control	04	0.648	0.2088	0.1044	0.31606	0.981	1.328	0.001
			Diseased	21	0.210	0.23	0.05	0.10	0.31	2.16	
		Δmeth	Control	06	2955.68	476.1	194.367	2456.04	3455.31	-0.301	0.52*
			Diseased	07	541.60	1911.98	722.660	-226.67	3309.88	0.377	
**TNM Stages**	Stage I	Expression	Control	36	0.499	1.019	0.169	0.155	0.844	4.559	0.310*
			Diseased	42	0.272	0.227	0.035	0.201	0.343	1.133	
		Δmeth	Control	36	1075.01	1138.52	189.75	689.79	1460.23	1.144	< 0.001
			Diseased	42	223.05	206.88	31.92	158.58	287.52	0.695	
	Stage II	Expression	Control	10	0.187	0.224	0.070	0.027	0.346	0.769	0.304*
			Diseased	13	0.233	0.188	0.052	0.119	0.347	0.515	
		Δmeth	Control	10	1551.67	1362.61	430.89	576.92	2526.42	0.103	0.619*
			Diseased	13	1206.23	934.55	259.199	641.48	1770.97	0.438	
	Stage III	Expression	Control	12	0.063	0.117	0.033	-0.010	0.138	1.438	0.013
			Diseased	19	0.090	0.085	0.019	0.049	0.132	0.893	
		Δmeth	Control	12	2912.96	1120.14	323.35	2201.253	3624.66	0.583	< 0.001
			Diseased	19	1419.51	1160.36	266.20	860.24	1978.79	0.753	
**Histological Grades**	DCI	Expression	Control	05	0.833	0.240	0.108	0.534	1.132	-0.41	0.008
			Diseased	06	0.220	0.154	0.063	0.058	0.382	0.392	
		Δmeth	Control	05	426.9	407.56	182.26	-79.08	933.00	0.755	0.784*
			Diseased	06	487.99	589.93	240.84	-131.10	1107.08	1.589	
	ILC	Expression	Control	11	0.189	0.327	0.098	-0.030	0.409	1.239	0.059
			Diseased	11	0.290	0.197	0.059	0.158	0.422	0.527	
		Δmeth	Control	11	1703.55	1714.26	516.87	551.896	2855.21	1.227	0.009
			Diseased	11	277.3	365.908	107.61	37.564	517.11	1.139	
	IDC	Expression	Control	33	0.41	1.050	0.182	0.039	0.784	4.821	0.021
			Diseased	33	0.313	0.283	0.049	0.212	0.414	1.278	
		Δmeth	Control	33	1558.59	1993.02	346.94	851.90	2265.29	2.782	0.013
			Diseased	33	784.95	1111.23	193.44	390.92	117.98	1.599	

*non-significant.

### Aberrant Promoter Methylation Around Rbl2/p130 TSS in Tumor Patients

**Fig 2:**
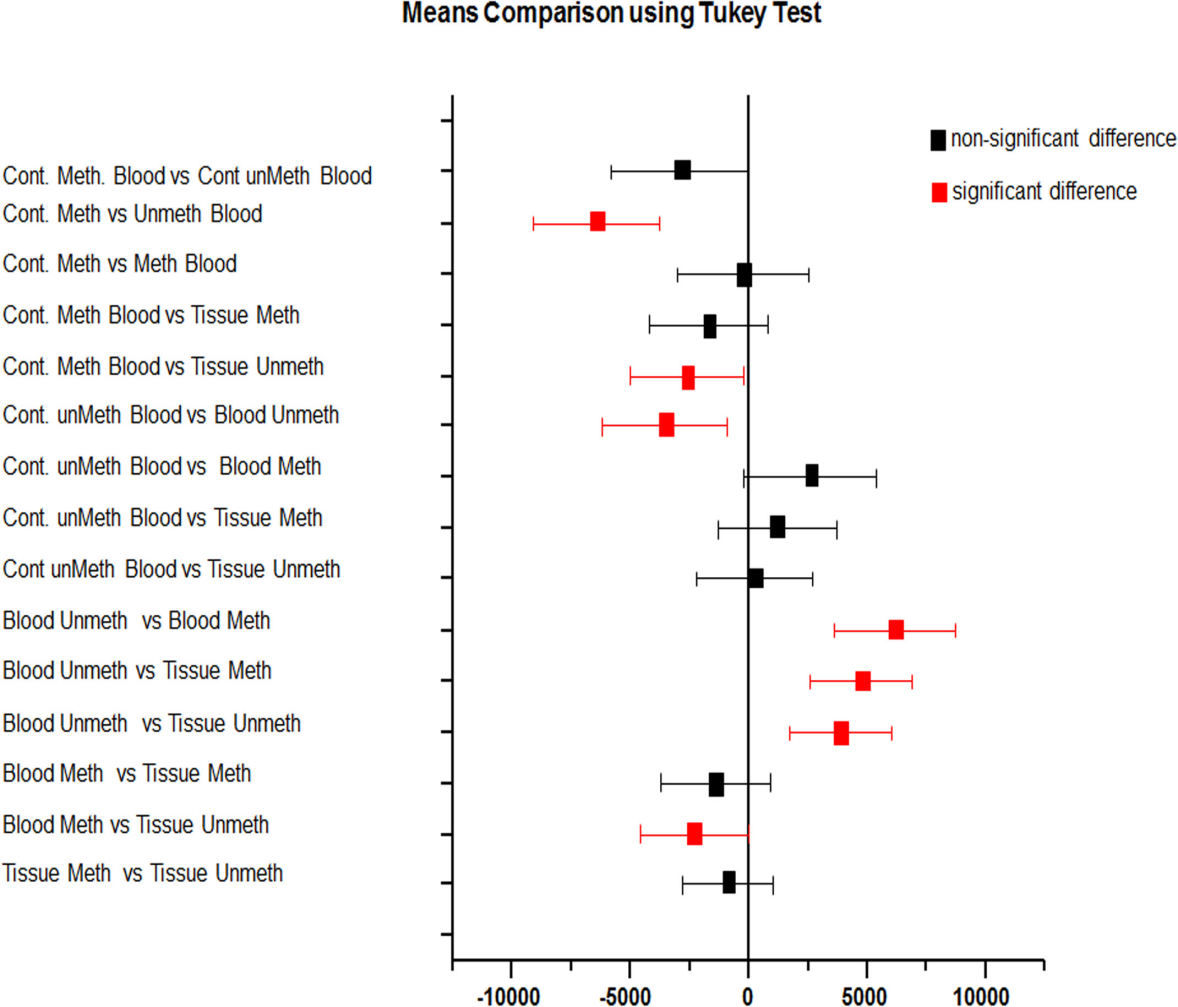
Mean comparison of Rbl2/p130 promoter methylation status using tukey test.

**Fig 3:**
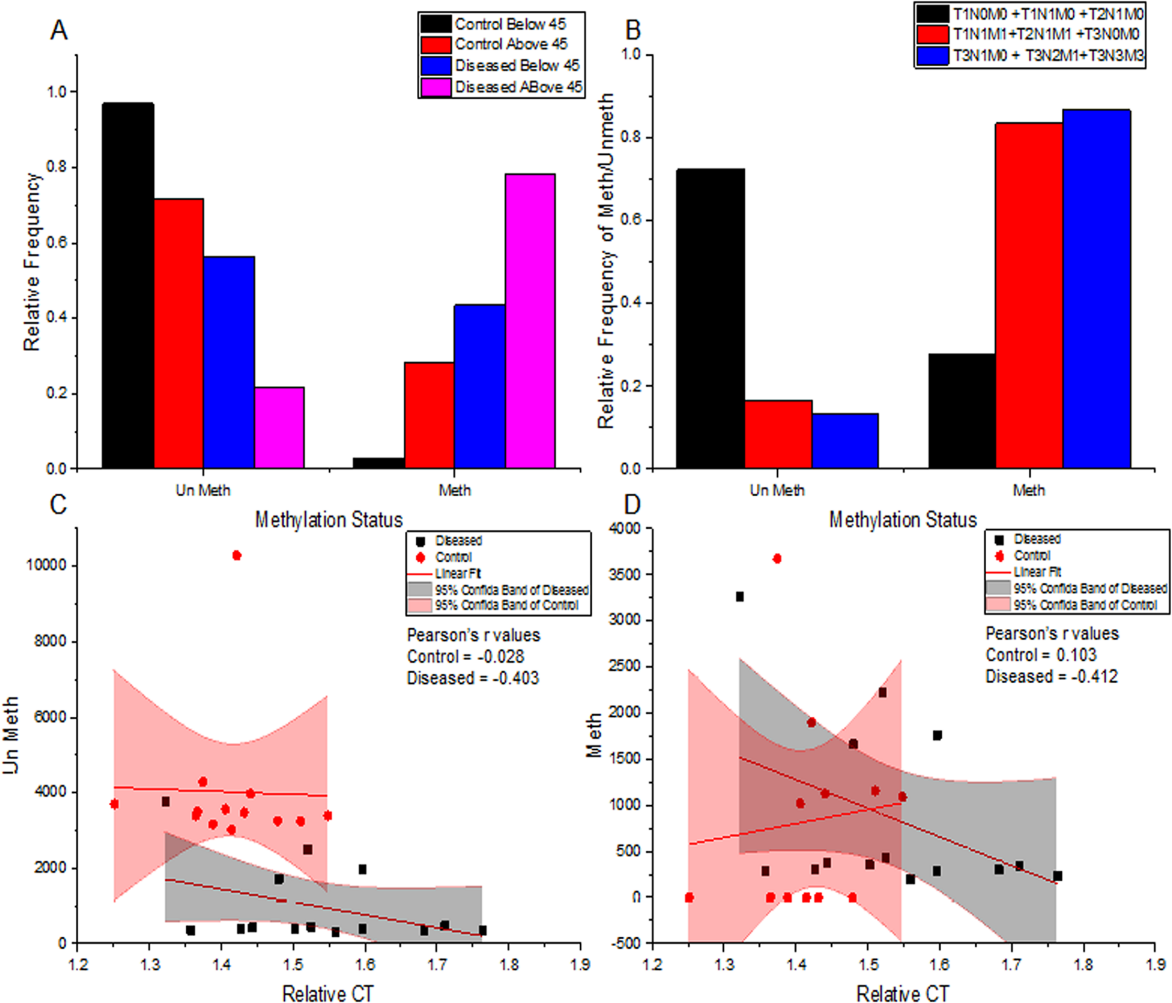
Statistical relation of Rbl2/p130 expression and its promoter methylation. (**A)** Differences in Rbl2/p130 promoter methylation status of tumor and normal tissues in study cohort I (<45 years) and study cohort II (≥45 years); (**B)** Observed differences in Rbl2/p130 promoter methylation status of various tumor tissues at different disease stages; (**C & D)** Correlation of relative expression (ΔCT values) and promoter methylation status as observed. Unmeth (C panel) and Meth (D panel) values are plotted separately. Adjusted R-values are shown in respective panels.

**Fig 4:**
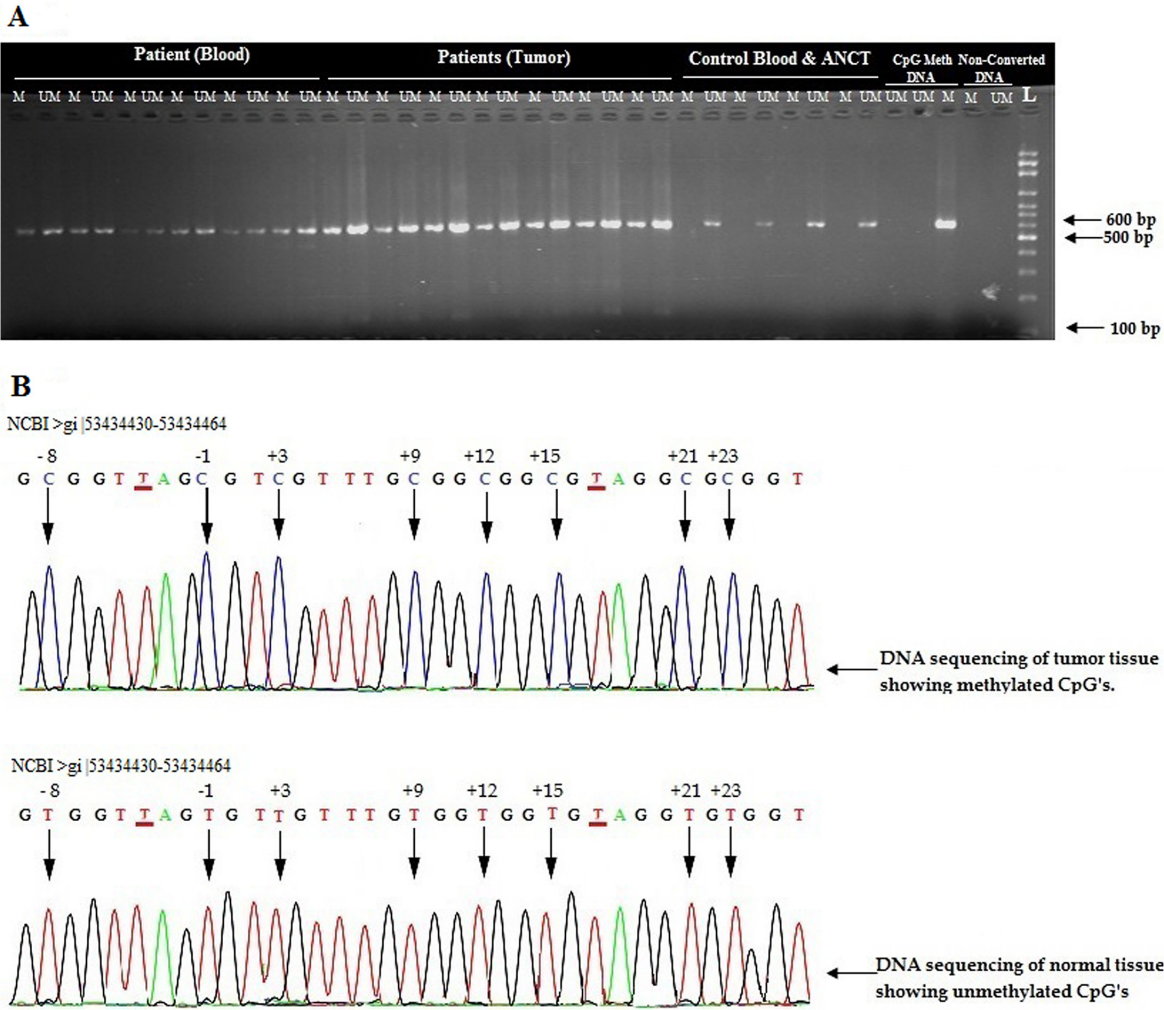
Promoter methylation analysis of Rbl2/p130 gene in normal individuals and breast cancer patients. **(A)** 2% agarose gel showing amplified products of methyl specific PCR (MSP). “M” represents methylation, “UM” represents un-methylation and L represents DNA size ladder. (**B)** Upper panel shows methylation of CpG “C” in tumors, whereas lower panel highlights un-methylation in control individuals. **T** shows position of non CpG “C” converted into **T** as a results of bisulfite conversion while arrows indicates the position of methylated cytosine at CpGs sites.

Promoter methylation status in breast tumors, adjacent normal tissues and blood from the same patients along with control blood samples were analyzed to correlate the effect of methylation with down regulation of RbL2/p130 gene in breast cancer patients (Fig 2). Total 80 CpG sites in upstream regulatory sequences (promoter) of RbL2/p130 (NCBI “53434362–53434967”), were targeted by methylation specific PCR. Methylation levels were found higher than un-methylation in cancer patients (Fig 3), as is also supported by gel quantitation results to measure relative band intensities (Table 3 and Fig 4A).

**Table 3:** Rbl2/p130 Promoter Methylation Status among Breast Cancer Patients.

Study Cohort	Samples Type	Frequency N (%)	Methylation N (%)	Un methylation N (%)
**< 45 years**	Control	33 (43.42)	02 (6.06)	31 (93.94)
	Tumor	33 (43.42)	8 (24.24)	25 (75.76)
**≥ 45 years**	Control	43 (56.58)	05 (11.63)	38 (88.37)
	Tumor	43 (56.58)	32 (74.42)	11 (25.58)
**Pre-menopausal**	Control	49 (64.47)	01 (2.04)	48 (97.96)
	Tumor	49 (64.47)	17 (34.69)	32 (65.31)
**Post-menopausal**	Control	27 (35.53)	06 (22.22)	21 (77.78)
	Tumor	27 (35.53)	23 (85.19)	04 (14.81)
**Type of Breast Cancer**	IDC	56 (73.68)	31(55.36)	25 (44.64)
	ILC	16 (21.06)	6 (37.5)	10 (62.5)
	DCI	4 (5.26)	3 (75.00)	1 (25.00)
**Clinical Stages**	Stage I, II	59 (77.63)	25 (42.37)	34 (57.63)
	Stage III	14 (18.42)	12 (85.71)	02 (14.29)
	Stage IV	03 (3.95)	03 (100)	00 (0.00)
**Tumor Grades**	Well Differentiated	34 (44.74)	06 (17.65)	28 (82.35)
	Moderate Differentiated	28 (36.84)	22 (78.57)	06 (21.43)
	Poor Differentiated	14 (18.42)	12 (85.71)	02 (14.29)

**Fig 5:**
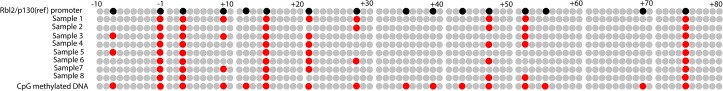
Map of Rbl2/p130 promoter showing methylated and un-methylated CpG’s cytosine ranging from −10 to +80 with reference to TSS (transcription start site).

Hyper methylation was observed in region (-10) to (+ 80) around transcription start site (TSS) of Rbl2/p130 gene. In general the CpG positions at (-1), (+3), (+15) and (+75) were found hyper-methylated in all samples. However the CpG positions (-8), (+9), (+21), (+28), (+47) and (+52) were found methylated in > 50% patients. In contrast, the CpG positions at (+12), (+35), (+39), (+43), (+55) and (+69) were observed un-methylated in all of the recruited patients. However in control (CpG methylated commercial Human DNA) reference sample all CpG site were found methylated running through targeted promoter region (Figs 4B and 5).

### Promoter Methylation Varies with Age Groups and Disease Status of Patients

Promoter methylation pattern was studied among different age and disease status individuals. Patients above the age of 45 years showed increased methylation level and decreased un-methylation status when compared with second study cohort i.e. participants <45 years of age (Fig 3A). The patients having <45 years ages showed higher un-methylation in ~76% of cases, while ~24% of the cases was methylated. In contrast the patients having ≥45 years ages were carrying higher levels of methylation (~74%) and lower level (~26%) of un-methylated promoter status. Moreover, Δmeth values were found to be significantly different (P = 0.019) in study cohort 1 i.e. <45 years (Tables 2 and 3). Similar observation was made for promoter methylation status for pre- and post-menopausal stages. In pre-menopausal stage ~35% cases showed higher methylation as compared to un-methylation (~65% cases), while patients with post-menopausal stage have higher methylation (in ~85% cases) compared to un-methylation (~15%) (Table 3).

**Fig 6:**
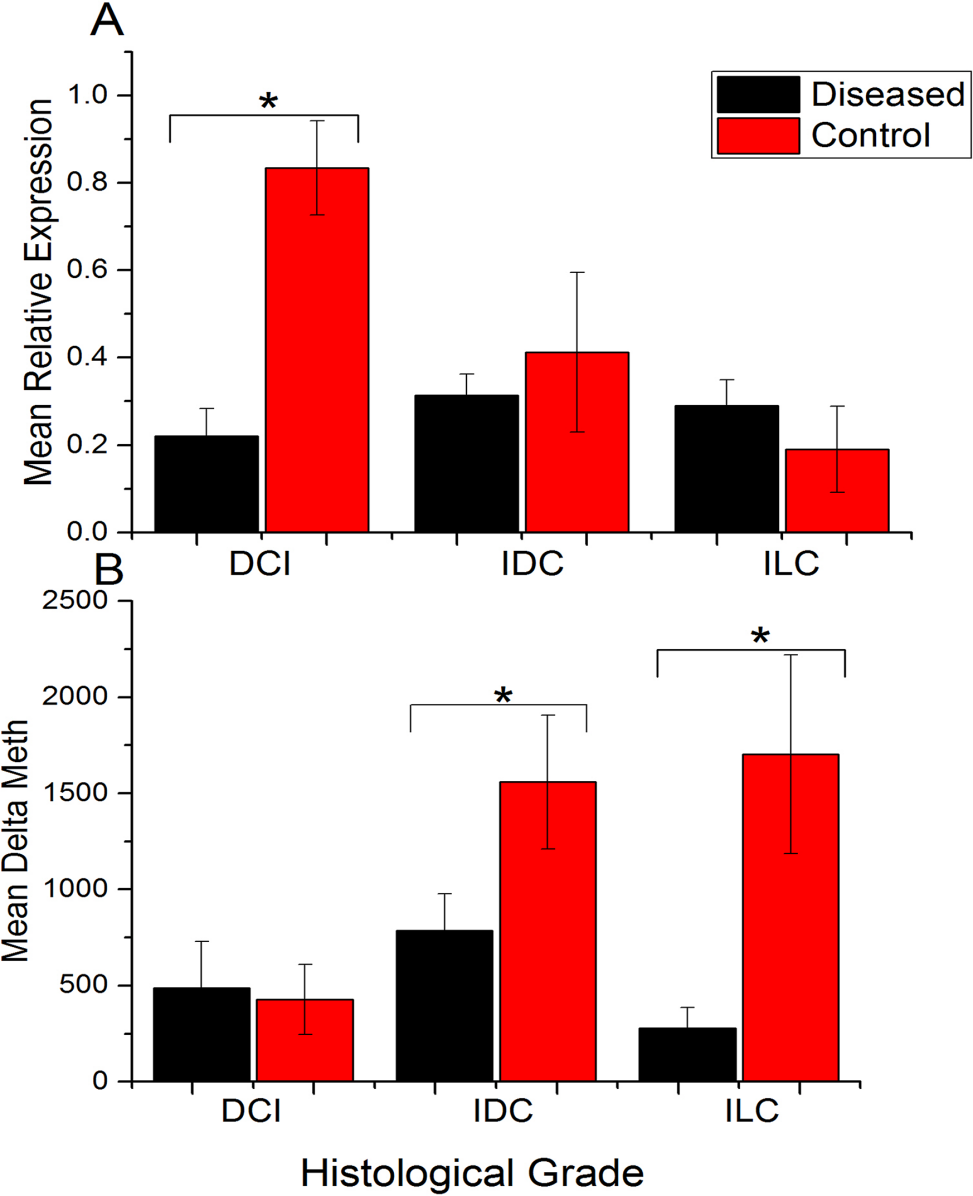
Rbl2/p130 promoter methylation and transcript expression analysis among various histological types of breast cancer. **(A)** Relative expression of Rbl2/p130 in control and tumor tissues among various histological types of breast cancer. **(B)** Change in promoter methylation (ΔMeth) status in control and diseased tissues among different histological different types of breast cancer. **DCI;** ductal carcinoma in situ. **IDC;** invasive ductal carcinoma. **ILC;** invasive lobular carcinoma.

Interestingly, levels of methylation were found to be increasing with advancing tumor stages, while their un-methylation status was decreasing (Fig 3B). Patients with disease stage I/II had lower methylation (~42% cases) compared to patients at advanced disease stage III, (~86%), while 100% methylation were observed patients at stage IV. Similarly, the high-grade tumors (poor differentiated) had higher methylation (~86%) status compared to moderate (~79%) and well-differentiated (~18%) tumor (Table 3). Among different histological types, patients with Ductal Carcinoma In Situ (DCI) show higher methylation frequency (75%) followed by Invasive Ductal Carcinoma (IDC ~55.36%) and Invasive Lobular Carcinoma (ILC ~37.5%) compared to un-methylation status as shown in Table 3. Statistical analysis revealed a significant difference among means (Δ meth) of disease and their adjacent normal control tissues in all histological tumor types except for DCI (Table 2 and Fig 6).

### Expression of Rbl2/p130 Negatively Correlates to Its Promoter Methylation

Methylation of cytosine at CpG sites around upstream regulatory (promoter) region of a gene may correspond to gene expression. It has been observed that the degree of methylation have significantly negative effect on expression level of Rbl2/p130 gene. Statistical analysis reveals a strong negative correlation between meth and un-meth levels with Rbl2 expression (r = -0.412, and r = -0.403 respectively) in breast cancer patients, whereas no significant correlation was found among Rbl2 expression and promoter methylation in control samples (Table 4). This reflects involvement of promoter methylation in regulation of Rbl2/p130 expression. In addition to that effect of aging on Rbl2 methylation status was also observed. Breast cancer patients showed strong negative correlation with meth and un-meth levels (~ r = -0.579), while control patients showed a weak negative correlation with age (Table 4 and Fig 3).

**Table 4:** Correlation Analysis of Various Study Parameters Used in Current Study.

Variable	Statistics Cohort	DF	Pearson's r	Adj. R-Square	F Value	P value
**Control**	Age vs Expression	11	0.206	-0.045	0.488	0.499
	Meth vs Expression	10	0.103	-0.088	0.108	0.749
	Unmeth vs Expression	11	-0.028	-0.090	0.009	0.927
	Meth vs Age	11	0.090	-0.082	0.089	0.771
	Unmeth vs Age	12	-0.196	-0.042	0.477	0.503
	DCI Δ meth vs DCI Expression	3	-0.55159	0.07234	1.31193	0.335
	ILC Δ meth vs ILC Expression	9	-0.27767	-0.02544	0.7519	0.408
	IDC Δ meth vs IDC Expression	31	-0.23179	0.0232	1.76012	0.194
**Diseased**	Age vs Expression	48	0.245	0.040	3.055	0.087
	Meth vs Expression	12	-0.412	0.100	2.452	0.143
	Unmeth vs Expression	12	-0.403	0.093	2.325	0.153
	Meth vs Age	12	-0.579	0.280	6.052	0.030
	Unmeth vs Age	12	-0.578	0.279	6.025	0.030
	DCI Δ meth vs DCI Expression	4	0.64373	0.26799	2.83052	0.168
	ILC Δ meth vs ILC Expression	9	-0.38488	0.05348	1.56499	0.242
	IDC Δ meth vs IDC Expression	31	0.13632	-0.01308	0.58696	0.449

## Discussion

Cytogenetically, p130 maps to human chromosomal area 16q12.2, an area repeatedly altered in human cancers [3, 5, 33, 34]. The Rbl2/p130 promoter shows a characteristic structural organization of "housekeeping" and growth control-related genes; a typical TATA or CAAT box is missing, but several GC rich zones and potential binding sites for numerous transcription factors are present [35].

Rbl2/p130 has been shown to block the cell cycle, whereas pRb overexpression sometimes fails to do so. Numerous evidences favor the role of Rbl2/p130 as a tumor suppressor protein. Rbl2/p130 down-regulates the cell cycle and it has also been shown to block the cell cycle[9]. Rbl2/p130 have been shown to inversely correlate with cancer malignancies by immune-histochemical analysis in endometrial carcinoma, oral squamous carcinoma and in uveal melanoma [5, 9, 36].

Our findings corroborate with these reports and tells another aspect of the same story; significant decrease (P = 0.001) in Rbl2/p130 expression in breast tumors tissues irrespective of patient’s age. This suggests that loss of Rbl2 expression is a frequent phenomenon in breast cancer pathogenesis. Furthermore Rbl2/p130 expression was drastically reduced to ~4 times in patients below the age of 45, although the down regulation in Rbl2/p130 expression was also present in patients above the age of 45 years (Fig 1D). Though our findings are in contradiction with an earlier study that reported decrease in Rbl2 expression in older patients than younger ones, the differences in Rbl2 expression among different ages groups in their study were statistically non-significant [37]. Moreover, their definition of younger age doesn’t correspond to ours, as they considered patients below 68 years as younger. Nevertheless, decrease in Rbl2/p130 expression seems to be an important event in tumor initiation among patients below the age of 45 years. Similarly our results also support previous assumption regarding loss of RbL2/p130 expression in age specific manner [15, 38]. Furthermore the frequency of Rbl2/p130 expression was significantly decreased with increasing tumor stage and grade (Tables 2 and 4 and Table A in S1 File). This highlights not only the significance of Rbl2/p130 expression as an early prognostic tool for breast cancer pathogenesis but also suggests its involvement in tumor progression. Loss of Rb2/p130 expression have been reported to be an important parameter for risk assessment of various cancers including non-small cell lung cancer progression [39, 40], endometrial and mammary carcinomas [37], ovarian cancers [15, 16] in their initial stages.

Several reports, not only discuss the loss of Rbl2/p130 in various types of cancer; but also give an insight on genetic alterations leading to silencing of Rbl2/p130 but epigenetic mechanisms involved in Rbl2/p130 silencing remains elusive. Although Rbl2/p130 has been shown to play role in ER-α promoter methylation by recruiting DNMTI at ER-α promoter [41], which might be an important event in deregulated expression of ER-α in various types of cancer, no comprehensive data is available on Rbl2/p130 promoter methylation itself. Here we report that aberrant promoter methylation at CpG sites around TSS of Rbl2/p130 might play a vital role in regulating its expression. DNA methylation is an important epigenetic phenomenon, responsible for enhanced expression of proto-oncogenes as well as silencing of tumor suppressor genes leading to abnormal proliferation and de-differentiation of cancer cells [28, 42].

Hyper-methylation in the region -10 to + 80 around TSS of Rbl2/p130 in tumor sample was observed. Interestingly these hyper methylated samples showed reduced Rbl2/p130 expression which further may lead the disease to metastasize. Previously it was investigated that the region of the retinoblastoma gene flanking over +13 to +18 near TSS is crucial for DNA methylation and thereby gene silencing [28]. It has been reported that aberrant promoter methylation may correspond to gene silencing and thus loss of function [43, 44]. Usually promoter methylation occur more frequently at CpG site located 5’UTR of genes [44]. It has been reported that DNA methylation of tumor suppressor gene increases twofold in stage III and IV thereby causing gene silencing in human esophageal squamous cell carcinoma as well as in breast cancer [21]. Our results reflect a strong negative correlation (Pearson coefficient r = -0.412; Table 4) among methylation status and Rbl2/p130 expression in diseased tissues. Circumstantial evidence supports the idea that aberrant promoter methylation might be the causative agent for reduced Rbl2/p130 expression and tumor initiation, and is worth exploring phenomenon as a new prognostic tool for breast cancer pathogenesis. This hypothesis is also supported by the fact that CpG cytocines at -1 and +3 positions around TSS were found hyper-methylated in almost all the samples. Intact TSS is required for efficient binding of the transcription machinery. Methylation in or around TSS might perturb this binding leading to reduced expression of Rbl2/p130. Similarly, 5’UTR (+15-+50) of the target candidate gene were found to be heavily methylated, has been reported previously for retinoblastoma protein (pRb) [28]. UTR play important role in transcript stability and it has been reported that 5’UTR play pivotal role to develop pathogenesis [45]. Similarly post transcriptional regulation of mRNA may also correspond to aberrant gene expression. Methylation in the promoter regions might affect the expression and stability of Rbl2/p130. Numerous single nucleotide polymorphisms (SNPs) have already been reported at 16q 12.2 region >gi|568815582: 53434362–53434967 flanking over TSS of Rbl2/p130. Interestingly, a CpG C >T (SNP “rs144092904” NCBI) conversion at -1 position of TSS has been reported. This signifies the importance of methylation at this position. This conversion protects fraction of the population to undergo methylation at this very crucial position. However no data is available on disease susceptibility for population with this SNP. Based on these findings, screening of C >T conversion in normal individuals is an interesting domain to explore. Therefore it has been concluded from this genetic and epigenetic characterization, that promoter methylation status may prove to be one of the important prognostic factor for the etiology of breast cancer.

## Ethical Standard

The study was conducted with a prior approval from the institutional ethical review board of COMSTAS Institute of Information Technology (CIIT) Islamabad. Members of this committee include Dean ORIC (Office of Research Innovation and Commercialization) Pro. Dr. Raheel Qamar (convener), Prof. Dr. Habib Bukhari (Chairman Deptt of Biosciences), Prof. Dr. Mahmood A Kayani and Dr. Tayyaba Yasmin (Associate Head of the Department). The ethical review board approved the execution of project entitled “Molecular Genetic & Epigenetic Characterization of Tumor Suppressor Rbl2/p130 in Human Breast Cancer”. Moreover all the samples were collected after a signed informed consent from the participants of the study.

## Supporting Information

S1 File Statistical Analysis of Rbl2/p130 Expression and Promoter Methylation Status in Breast Cancer Patients. Rbl2/p130 expression and its promoter methylation status are significantly different among various stages of breast cancer patients. **Table A.** Statistical Analysis of Rbl2/p130 Transcript Expression and its Promoter Methylation Status among Control and Diseased Categories. **Table B.** Statistical Analysis of Rbl2/p130 Expression among Breast Cancer Patients.
(DOCX)
