# Supplementary material for: Promoter Methylation Status Modulate the Expression of Tumor Suppressor (RbL2/p130) Gene in Breast Cancer
Source: PLoS One. 2015 Aug 13;10(8):e0134687. doi: 10.1371/journal.pone.0134687 (PMC4536218; doi:10.1371/journal.pone.0134687)
Supplement: S1 File — Rbl2/p130 expression and its promoter methylation status are significantly different among various stages of breast cancer patients. Table A. Statistical Analysis of Rbl2/p130 Transcript Expression and its Promoter Methylation Status among Control and Diseased Categories. Table B. Statistical Analysis of Rbl2/p130 Expression among Breast Cancer Patients. (DOCX) [file pone.0134687.s001.docx]

| **Variables** | **Samples** | **Analysis** | **Category** | **N** | **Mean** | **SD** | **SEM** | **95%CI of Mean** | | **Skewness** | **P value** |
| --- | --- | --- | --- | --- | --- | --- | --- | --- | --- | --- | --- |
|  |  |  |  |  |  |  |  | **Lower** | **Upper** |  |  |
| **TNM Stages** | Diseased | Expression | Stage I | 42 | 0.273 | 0.227 | 0.035 | 0.202 | 0.344 | 1.133 | 0.005 |
|  |  |  | Stage II | 13 | 0.233 | 0.189 | 0.052 | 0.119 | 0.347 | 0.515 |  |
|  |  |  | Stage III | 19 | 0.090 | 0.085 | 0.019 | 0.050 | 0.132 | 0.892 |  |
|  |  | ∆meth | Stage I | 42 | 223.056 | 206.889 | 31.923 | 158.585 | 287.527 | 0.695 | < 0.001 |
|  |  |  | Stage II | 13 | 1206.231 | 934.555 | 259.199 | 641.484 | 1770.977 | 0.438 |  |
|  |  |  | Stage III | 19 | 1419.519 | 1160.368 | 266.206 | 860.240 | 1978.799 | 0.753 |  |
|  | Control | Expression | Stage I | 36 | 0.499 | 1.018 | 0.170 | 0.155 | 0.844 | 4.559 | 0.226* |
|  |  |  | Stage II | 10 | 0.187 | 0.223 | 0.071 | 0.027 | 0.347 | 0.768 |  |
|  |  |  | Stage III | 12 | 0.063 | 0.117 | 0.033 | -0.010 | 0.138 | 1.438 |  |
|  |  | ∆meth | Stage I | 36 | 1075.016 | 1138.524 | 189.754 | 689.795 | 1460.237 | 1.144 | < 0.001 |
|  |  |  | Stage II | 10 | 1551.673 | 1362.610 | 430.895 | 576.920 | 2526.426 | 0.103 |  |
|  |  |  | Stage III | 12 | 2912.960 | 1120.146 | 323.358 | 2201.253 | 3624.667 | 0.583 |  |
| **Histological Grades** | Diseased | Expression | DCI | 6 | 0.220 | 0.154 | 0.063 | 0.058 | 0.382 | 0.392 | 0.712* |
|  |  |  | ILC | 11 | 0.290 | 0.197 | 0.059 | 0.158 | 0.422 | 0.527 |  |
|  |  |  | IDC | 33 | 0.313 | 0.284 | 0.049 | 0.213 | 0.414 | 1.040 |  |
|  |  |  | DCI | 6 | 487.991 | 589.938 | 240.841 | -131.105 | 1107.089 | 1.589 | 0.293* |
|  |  |  | ILC | 11 | 277.339 | 356.908 | 107.612 | 37.564 | 517.113 | 1.140 |  |
|  |  |  | IDC | 33 | 784.954 | 1111.239 | 193.441 | 390.925 | 1178.982 | 1.599 |  |
|  | Control | Expression | DCI | 5 | 0.833 | 0.241 | 0.108 | 0.534 | 1.133 | 1.278 | 0.414* |
|  |  |  | ILC | 11 | 0.190 | 0.327 | 0.099 | -0.030 | 0.409 | 1.239 |  |
|  |  |  | IDC | 33 | 0.412 | 1.050 | 0.183 | 0.040 | 0.784 | 4.821 |  |
|  |  | ∆meth | DCI | 5 | 426.960 | 407.568 | 182.270 | -79.083 | 933.005 | 0.755 | 0.405* |
|  |  |  | ILC | 11 | 1703.556 | 1714.267 | 516.871 | 551.896 | 2855.217 | 1.227 |  |
|  |  |  | IDC | 33 | 1558.598 | 1993.023 | 346.941 | 851.903 | 2265.294 | 2.783 |  |

**Table A** **Statistical Analysis of Rbl2/p130 Transcript Expression and its Promoter Methylation Status among Control and Diseased Categories**

* non-significant

**Table B Statistical Analysis of Rbl2/p130 Expression among Breast Cancer Patients.**

| **Study Variables** | | **N** | **Mean** | **SD** | **SEM** | **95% CI of Mean** | | **Skewness** | **P value** |
| --- | --- | --- | --- | --- | --- | --- | --- | --- | --- |
|  | |  |  |  |  | Lower | Upper |  |  |
| **Overall** | Control | 13 | 1.00 | 1.52 | 0.42 | 0.08 | 1.92 | 3.34 | 0.001 |
|  | Diseased | 50 | 0.28 | 0.24 | 0.04 | 0.21 | 0.35 | 1.64 |  |
| **Age-wise** | ≥ 45 | 21 | 0.21 | 0.23 | 0.05 | 0.10 | 0.31 | 2.16 | 0.011 |
|  | < 45 | 29 | 0.36 | 0.25 | 0.05 | 0.27 | 0.46 | 1.13 |  |
| **TNM Stages** | Stage 1 | 11 | 0.20 | 0.11 | 0.03 | 0.13 | 0.28 | -0.89 | 0.022 |
|  | Stage 2 | 15 | 0.24 | 0.23 | 0.06 | 0.12 | 0.37 | 1.00 |  |
|  | Stage 3 | 22 | 0.34 | 0.29 | 0.06 | 0.21 | 0.47 | 1.48 |  |
| **Histological Grades** | DCI | 6 | 0.22 | 0.15 | 0.06 | 0.06 | 0.38 | 0.39 | 0.713* |
|  | IDC | 33 | 0.31 | 0.28 | 0.05 | 0.21 | 0.41 | 1.28 |  |
|  | ILC | 11 | 0.29 | 0.20 | 0.06 | 0.16 | 0.42 | 0.53 |  |

* non-significant
